# Nuclear FABP7 immunoreactivity is preferentially expressed in infiltrative glioma and is associated with poor prognosis in EGFR-overexpressing glioblastoma

**DOI:** 10.1186/1471-2407-6-97

**Published:** 2006-04-19

**Authors:** Yu Liang, Andrew W Bollen, Ken D Aldape, Nalin Gupta

**Affiliations:** 1 Department of Neurological Surgery, Brain Tumor Research Center, University of California, San Francisco, CA 94143, USA; 2 Current address: Division of Molecular Biology, Sequence Detection System & Arrays, Applied Biosystems, Foster City, CA 94404, USA; 3 Department of Pathology, University of California, San Francisco, CA 94143, USA; 4 Department of Pathology, Section of Neuropathology, University of Texas M. D. Anderson Cancer Center, Houston, TX 77030, USA

## Abstract

**Background:**

We previously identified brain type fatty acid-binding protein (FABP7) as a prognostic marker for patients with glioblastoma (GBM). Increased expression of FABP7 is associated with reduced survival. To investigate possible molecular mechanisms underlying this association, we compared the expression and subcellular localization of FABP7 in non-tumor brain tissues with different types of glioma, and examined the expression of FABP7 and epidermal growth factor receptor (EGFR) in GBM tumors.

**Methods:**

Expression of FABP7 in non-tumor brain and glioma specimens was examined using immunohistochemistry, and its correlation to the clinical behavior of the tumors was analyzed. We also analyzed the association between FABP7 and EGFR expression in different sets of GBM specimens using published DNA microarray datasets and semi-quantitative immunohistochemistry. *In vitro *migration was examined using SF763 glioma cell line.

**Results:**

FABP7 was present in a unique population of glia in normal human brain, and its expression was increased in a subset of reactive astrocytes. FABP7 immunoreactivity in grade I pilocytic astrocytoma was predominantly cytoplasmic, whereas nuclear FABP7 was detected in other types of infiltrative glioma. Nuclear, not cytoplasmic, FABP7 immunoreactivity was associated with EGFR overexpression in GBM (N = 61, p = 0.008). Expression of the *FABP7 *gene in GBM also correlated with the abundance of *EGFR *mRNA in our previous microarray analyses (N = 34, p = 0.016) and an independent public microarray dataset (N = 28, p = 0.03). Compared to those negative for both markers, nuclear FABP7-positive/EGFR-positive and nuclear FABP7-positive/EGFR-negative GBM tumors demonstrated shortest survival, whereas those only positive for EGFR had intermediate survival. EGFR activation increased nuclear FABP7 immunoreactivity in a glioma cell line *in vitro*, and inhibition of FABP7 expression suppressed EGF-induced glioma-cell migration. Our data suggested that in EGFR-positive GBM the presence of nuclear FABP7 immunoreactivity increases the risk of poor prognosis

**Conclusion:**

In this study, we identified a possible mechanism as the basis of the association between nuclear FABP7 and poor prognosis of GBM. FABP7 expression can be found in all grades of astrocytoma, but neoplastic cells with nuclear FABP7 were only seen in infiltrative types of tumors. Nuclear FABP7 may be induced by EGFR activation to promote migration of GBM tumor cells. Positive nuclear FABP7 and EGFR overexpression correlated with short survival in EGFR-positive GBM patients. Therefore, nuclear FABP7 immunoreactivity could be used to monitor the progression of EGFR-overexpressed GBM.

## Background

GBM is the highest grade of astrocytoma and is also the most common primary brain tumor in adults. Approximately 50% of patients with GBM die within a year of diagnosis, despite the use of many aggressive treatment approaches [1]. Lack of reliable prognostic markers for these patients is a hindrance to improving therapy and individualizing therapeutic interventions. Amplification and/or overexpression of the *EGFR *gene, mutation of the *p53 *gene, and proliferation indices have all been proposed to predict survival of patients with GBM and to play a role in the pathophysiology of their tumors [2,3]; however, other studies have shown no such association with outcome [4-6]. One reason for this discrepancy is that strong clinical factors such as patient age need to be included [7,8]. Although clinical parameters such as age, Karnofsky performance status at diagnosis, and extent of resection are routinely used in clinical practice to predict the outcome of patients with GBM, none of these variables have a direct connection with tumor pathogenesis.

In a previous study, gene expression profiling of a group of GBM specimens identified a cluster of about 50 named genes whose expression was inversely associated with survival [9]. In examining the annotations of "biological process" in the Gene Ontology terms for each gene [10], the annotation "neurogenesis" appeared most frequently, suggesting a common role for these genes in central nervous system development. In contrast, a number of other annotations for biological process such as "cell proliferation," "inflammatory response," and "immune response" were underrepresented in these genes. Because several of these genes are involved in cell-cell and cell-matrix interactions and cell migration, we hypothesized that their increased expression might be related to more infiltrative and aggressive tumor behavior. Based on the results of the preceding analyses and the availability of antibodies, we chose to investigate the prognostic value of one gene, *FABP7*, in greater detail [9].

Although FABP7 is a cytoplasmic protein, its varying subcellular localization between nucleus and cytoplasm has been reported in developing brain [11], glioma cell lines [12], and GBM specimens [9]. Increased FABP7 expression was also found in glia following nerve injury [13,14]. We separately scored FABP7 immunoreactivity in nucleus and cytoplasm, and found that nuclear FABP7 immunoreactivity was inversely correlated with survival of patients with GBM, particularly in younger cases [9]. This result is consistent with other reports that emphasize the effect of age upon various prognostic factors [7,8], and such a pattern is similar to a recent finding of an association between EGFR overexpression and poor prognosis in younger GBM patients [7].

FABP7 is a member of the multi-gene fatty acid-binding protein (FABP) family and binds to very-long-chain polyunsaturated fatty acids (C > 16) such as docosahexaenoic acid (DHA) with very high affinity *in vitro *[15]. FABP7 appears to have different roles in different tissue types. It is highly expressed in radial and Bergmann glial cells throughout the developing central nervous system and gradually declines in the adult [16,17]. FABP7 is required for neuron-induced glial differentiation and subsequent migration of neurons along the glial processes, but has no effect on cell proliferation and adhesion [11]. In Schwann cells, FABP7 expression is downstream of the Ras-independent EGFR signaling pathway, and it regulates interactions between Schwann cells and axons in normal peripheral nerves and peripheral nerve tumors without affecting cell proliferation and migration [13]. Differential expression of different types of FABPs is found in several types of tumors and their normal-cell counterparts, and FABPs have been shown to modulate growth and differentiation of normal and neoplastic cells [18]. FABP7 was also shown to induce mammary differentiation and to mediate growth inhibition of breast cancer cells [19,20].

It has been suggested that FABPs increase the solubility of fatty acids in the cytoplasm when transporting fatty acids between membrane compartments, and bring fatty acids to their nuclear targets [21]. In addition, subcellular localization of FABPs appears to be tissue specific and closely associated with gene expression and function. Liver-type FABP targets fatty acids to the nucleus and interacts with peroxisome proliferator-activated receptors α and γ to regulate gene expression [22-24]. Soluble FABP7 was found to induce differentiation of the mammary gland in mice [20]. FABP7 is present in both the nucleus and cytoplasm in glial cells, as well as in the conditioned media of glial cells in culture [11], but is found only in the cytoplasm of Kupffer cells in the liver [25].

In this study, we examined the expression patterns and subcellular localization of FABP7 in specimens from normal individuals, from individuals with gliosis only, and from patients with gliomas differing in grade and histology. We also used independent sets of GBM specimens to analyze the relationship of subcellular localization of FABP7 with patient outcome and EGFR expression, and to seek possible mechanisms underlying the functions of FABP7 in GBM.

## Methods

### Cell culture

Glioma cell lines were obtained from the Neurosurgery Tissue Bank at the University of California, San Francisco. Immortalized human astrocytes were provided by Dr. Russ Pieper (University of California, San Francisco) [26]. All cells were maintained in Eagle's minimal essential medium with 10% FBS and 5% CO_2_.

### Tissue specimens

Frozen and paraffin-embedded specimens were obtained from the Neurosurgery Tissue Bank at the University of California, San Francisco, and University of Texas, M. D. Anderson Cancer Center after approval from the Committee on Human Research. Tissue sections for immunohistochemistry were of 5 μm in thickness. Gliotic and normal brain tissues were obtained from epileptic patients and postmortem specimens, respectively. Clinical data of patients with primary GBM that were used for analyzing the correlation between FABP7 and EGFR immunoreactivity are summarized in Tables 2 and 4. Two cohorts of GBM patients (61 and 44 cases, respectively) for EGFR expression analysis are the same sets used in a previous study [9].

### Antibodies

FABP7-specific polyclonal antibodies were gifts from Drs. N. Heintz (Rockefeller University, New York, NY) and R. Godbout (University of Alberta, Alberta, Canada). Antibodies from both sources produced similar staining patterns and specificity on Western blots and immunohistochemistry using GBM specimens ([12] and data not shown). A dilution of 1 to 400 was used for both immunostaining and migration assays, and a dilution of 1 to 500 was used for immunoblotting; secondary antibodies alone did not show detectable signal. Dilution of antibodies against glial fibrillary acidic protein (GFAP) (ICN; Costa Mesa, CA) and EGFR (clone F4; Sigma, St. Louis, MO) for immunohistochemistry was 1:1000 and 1:400, respectively. Peroxidase-conjugated and biotinylated secondary antibodies were obtained from Vector Laboratories (Burlingame, CA). Fluorescine-conjugated and Rhodamine-conjugated secondary antibodies and normal rabbit serum were obtained from Jackson ImmunoResearch Laboratories (West Grove, PA).

### Western blot analysis

Total RNA was extracted from frozen tissues specimens using Trizol (Invitrogen; Carlsbad, CA) as described previously [9], and genomic DNA was removed from the interphase and organic phase by ethanol precipitation. The protein fraction was then purified by isopropanol precipitation, washed several times in 0.3 M guanidine hydrochloride in 95% ethanol, and resuspended in 1% SDS. The protein concentration of each sample was quantitated by using a D_c _Protein Assay Kit (Bio-Rad; Hercules, CA), and equal amounts of protein for each sample were separated by SDS-PAGE and transferred to nitrocellulose membranes (Bio-Rad), blocked with 10% skim milk, incubated with specific antibodies, and visualized using a Super Signal West Pico Chemiluminescent kit (Pierce; Rockford, IL).

### Immunohistochemistry

All frozen tissue sections used for immunohistochemistry were fixed in 4% formaldehyde, treated with H_2_O_2_, blocked with normal serum, incubated with primary antibodies at 4°C overnight or room temperature (RT) 2 hours, incubated with biotinylated secondary antibody and peroxidase-labeled streptavidin at RT for 30 min, to visualize the immunoreactivity with the DAB Reagent kit (KPL; Gaithersburg, MA). Staining of paraffin-embedded sections followed the same protocol, except for prior de-waxing and antigen retrieval by microwave heating. Immunostaining and semi-quantitative scoring of p53 and EGFR expression were performed as previously described [7].

SF763 glioma cells were plated and incubated overnight in Lab-Tek chamber slides (Nalge Nunc International; Rochester, NY) followed by 24 hours of 0.5% serum starvation and 2 days of 50 ng/ml EGF (Invitrogen) treatment. After fixation in 4% formaldehyde, cells were blocked with normal serum, followed by 2 hours of RT incubation with the primary antibody and 1 hour of RT incubation with the secondary antibody. Cells were then covered with Vectashield (Vector Laboratories) to prevent fading of fluorescence. The fluorescence intensity of 200 control or EGF-treated cells was digitally recorded and the ratios were calculated.

### Antisense inhibition and migration assay

Antisense oligodeoxynucleotides (ODNs) used were complementary to the position -13 to 7 of the FABP7 cDNA, and sense ODNs complementary to the same region were used as a control. The first 3 and the last 3 phosphodiester bonds on the ODNs were modified to phosphorothioate bonds to prevent degradation. SF763 glioma cells were serum-starved in 0.5% serum-containing medium for 24 hours, followed by 2 days of 50 ng/ml EGF treatment in the same low-serum media. Control cells were maintained in the low-serum medium for 2 days. ODNs were incubated with FuGene (Roche, Basel, Switzerland) at RT for 30 min, and then added to SF763 cells on the second day of EGF treatment to a final concentration of 100 nM.

The inserts of TransWell chambers (Corning, Corning, NY) with 5μm pores were incubated with 100 μg/ml of rat-tail type 1 collagen (BD Biosciences, San Jose, CA) overnight at room temperature, and washed with phosphate-buffered saline (PBS). At the end of the 2-day EGF treatment, cells were dislodged using 2 mM EDTA in PBS and then resuspended in the same treatment media as before (control or EGF, sense or antisense ODNs). Low-serum medium was placed in the bottom well, and 1 × 10^4 ^cells were plated into each insert. After 4 hours, un-migrated cells were removed with cotton swabs and migrated cells were fixed and stained using a HEMA 3 stain set (Fisher Diagnostics, Middletown, VA). For each insert, cell numbers were counted from five randomly chosen fields under 200× magnification.

### Data analysis

All statistical analyses used SPSS for Windows (Release 11.5.0). The fluorescence intensity recorded using the Openlab software (Improvision, Lexington, MA) and migration data were analyzed using Student's t test. Correlation of nuclear localization of FABP7 with patient survival was analyzed using the Cox proportional hazards regression. Hazard ratios provide information about the direction of an association (a numeral over 1 indicates an increased risk with the positive variable, and a numeral under 1 indicates a decreased risk) as well as the magnitude of the risk. To evaluate the relationship between nuclear FABP7 and EGFR upon patient survival, new variables were used to divide patients into four groups based on the immunoreactivity of nuclear FABP7 and EGFR of their tumors: dual negative as "0", nuclear FABP7-negative/EGFR-positive as "1", nuclear FABP7-positive/EGFR-negative as "2", and dual positive as "3". Bivariate correlations were evaluated by the Spearman test. A p value < 0.05 was considered statistically significant for all tests.

## Results

### Differential glial fibrillary acidic protein immunoreactivity identifies four subsets of fatty acid-binding protein 7-expressing cells in adult normal and gliotic brain tissues

**Figure 1:**
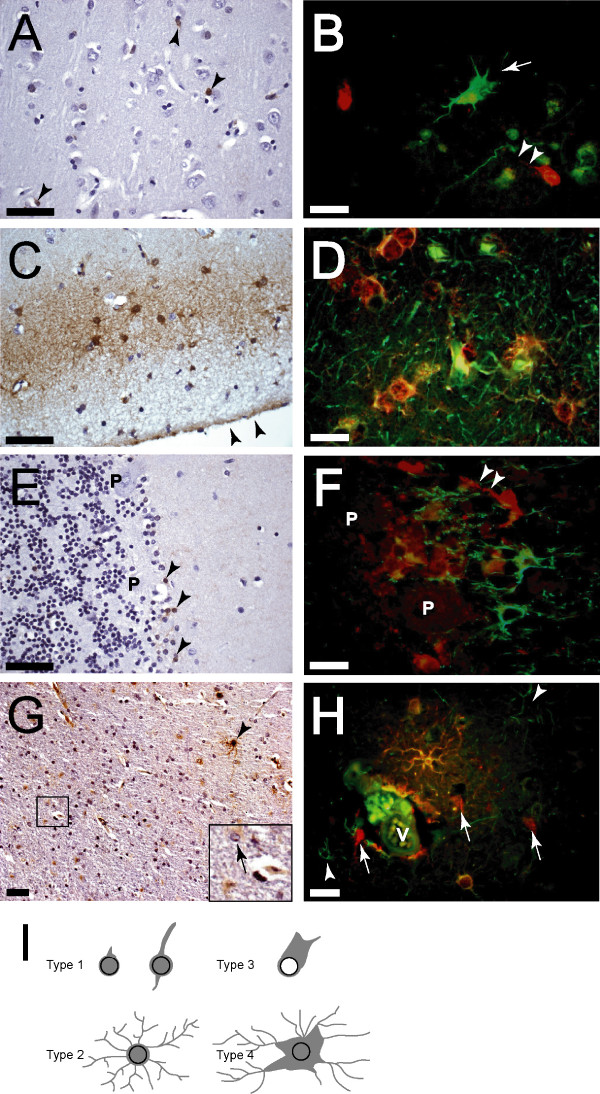
Several groups of glial cells in adult normal and gliotic brains expressed FABP7. *A*, scattered FABP7-positive Type 1 cells (arrowheads) were detected in normal cerebral cortex. Nuclei of these cells were irregular or elongated, and appeared to be larger than those of oligodendrocytes. *B*, FABP7-positive Type 1 cells usually had only one or two processes (arrowheads), and were distinctive from GFAP-expressing astrocytes bearing elaborate processes (arrow). *C *and *D*, all FABP7-positive Type 2 cells localized at the subpial layer had numerous processes and expressed GFAP. Arrowheads in *C *indicate pia. Some GFAP+/FABP7- astrocytes could be seen. *E*, scattered FABP7-positive Type 1 cells were detected in normal cerebellum (arrowheads), whereas granule cells and Purkinje cells (P) were FABP7 negative. *F*, FABP7 and GFAP staining identified two distinctive populations of astrocytes. Arrowheads indicate the process of a FABP7-positive Type 1 cell. *G*, the number of FABP7-positive cells was increased in gliotic tissues, and Types 3 (arrow in inset) and 4 (arrowhead) cells appeared. *H*, in a region of perivascular gliosis, several GFAP+/FABP+ reactive astrocytes (Type 4) were seen. There were also FABP7-negative reactive astrocytes (arrowheads) and Type 1 cells (arrows) in this region. Strong immunofluorescence inside the vessel (V) was autofluorescence. *I *is a schematic summary of the morphology of the four types of glial cells identified in normal and gliotic adult brains. Gray color indicates FABP7 immunoreactivity. Note that Type 3 cells have gemistocytic characteristics and usually do not have nuclear FABP7. Bars in *A*,*C*,*E*,*G*, 50μm; bars in *B*, *D*, *F*, *H*, 20μm, and red and green fluorescence represents FABP7 and GFAP, respectively.

We compared expression and subcellular localization of FABP7 in adult normal and gliotic brain specimens using immunohistochemistry. In normal cerebrum, there was minimal FABP7 reactivity in the white matter (data not shown), and only scattered, predominantly nuclear FABP7 immunoreactivity in the cortex (Fig. 1A). Morphologically, the positive cells appeared to be glial in origin but were negative for GFAP. These cells had a thin rim of cytoplasm with no processes or with much fewer processes than their GFAP-positive counterparts (Fig. 1B). For purpose of comparison, we named this unique group of cells Type 1 cells. A separate population of GFAP-positive cells in the subpial layer was also found to be positive for FABP7 (Fig. 1C and 1D). We designated these cells Type 2 cells; these were distinguishable from Type 1 cells by their location, elaborate processes, GFAP immunoreactivity, and FABP7 staining throughout their nucleus, cytoplasm, and processes. The presence of Type 2 cells in the subpial layer is consistent with a previous finding in which FABP7 mRNA was found in the glia limitans of adult mice [27]. Endothelial cells and neurons were negative for FABP7 expression (Fig. 1A, and additional data not shown). In the cerebellum, only scattered FABP7-positive glial cells were identified, mostly in region of the Purkinje cell layer (Fig. 1E), which is also in agreement with a previous *in situ *hybridization study in mice [27]. These positive cells were very similar to the Type 1 cells we identified in cerebral cortex (Fig. 1F).

The expression of FABP7 and the number of FABP7-positive cells were increased in the cortex of gliotic brain specimens, although the subcellular localization of FABP7 was variable (Fig. 1G). In addition to the Type 1 cells, two distinct types of FABP7-positive cells, designated as Type 3 and Type 4 cells, were identified in gliotic cerebral cortex, and were probably reactive astrocytes. Type 3 cells showed FABP7 immunoreactivity mainly in their gemistocytic cytoplasm, while nuclear staining was absent (Fig. 1G, inset). Type 4 cells featured hypertrophic cytoplasm and elaborate processes, and their nucleus, cytoplasm, and processes are strongly positive for FABP7 (Fig. 1G). Both Type 3 and Type 4 cells expressed GFAP (Fig. 1H), and were not found in normal cerebral cortex, but only a subset of reactive astrocytes expressed FABP7 (Fig. 1H). Another feature in gliotic brain specimens was that FABP7 was weakly positive in the cytoplasm of some neurons (data not shown). Figure 1I summarizes the morphology of the four types of FABP7-expressing glial cells described above.

### Nuclear fatty acid-binding protein 7 is detected in neoplastic astrocytes in infiltrative types of glioma but not in grade I pilocytic astrocytoma

Because FABP7 demonstrates increased expression and variable subcellular localization in a subset of reactive astrocytes but not in most oligodendrocytes, we determined whether FABP7 is universally expressed in different grades of astrocytoma. For comparison, we also examined specimens from oligoastrocytoma (OAC) and oligodendroglioma (ODG).

**Figure 2:**
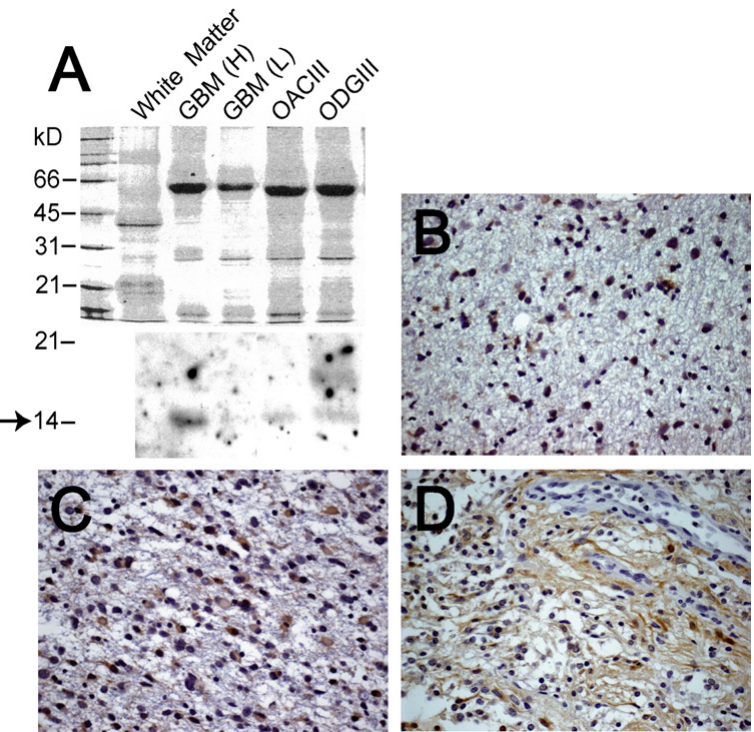
FABP7 was detected in all grades of astrocytomas. *A*, *lower*, expression of FABP7 was analyzed Western blotting using lysate from normal white matter, two GBM specimens (H and L indicate high and low, respectively (high and low FABP7 expression based on the results of the previous microarray study [9]), one oligoastrocytoma grade III (OACIII), and one oligodendroglioma grade III (ODGIII); *upper*, the protein loading was demonstrated by Coomassie Blue staining. The arrow indicates the size of the FABP7 core protein. The 14 kD band in GBM (L) appeared after longer exposure of the film. The 18 kD band that appeared in the ODGIII lysate may not be non-specific, since several immunoreactive bands in that region could also be detected in lysate from a panel of glioma cell lines (data not shown). Representative photomicrographs of immunohistochemistry of FABP7 in grade II astrocytoma (*B*), grade III anaplastic astrocytoma (*C*), and grade I pilocytic astrocytoma (*D*) demonstrated distinctive FABP7 immunoreactivity in the cytoplasm and cell processes in pilocytic astrocytomas as opposed to the nuclear and cytoplasmic staining in grades II and III astrocytomas. FABP7-positive nuclei that were occasionally detected resembled the Type 1 cells seen in normal brain. The scale of the photomicrographs is the same as in Figure 1 *A*.

An immunoblot of extracts from two GBM specimens and two oligodendroglial tumors showed detectable levels of FABP7 protein (Fig. 2A). We found that 1 of 5 grade II astrocytoma specimens and 3 of 5 grade III astrocytoma specimens were positive for FABP7. Paralleling the results obtained from GBM specimens [9], both nuclear and cytoplasmic FABP7 immunoreactivity was found in these tumors (Fig. 2B and 2C). FABP7-positive reactive astrocytes similar in morphology to the Types 3 and 4 cells (data not shown) were observed occasionally. We also examined 10 pilocytic astrocytoma specimens (grade I), and, in agreement with previous gene expression profiling of this type of tumor [28], 9 of our specimens were positive for FABP7. Interestingly, the FABP7 immunoreactivity in these 9 cases was predominantly in the cytoplasm and cell processes, not in the nuclei (Fig. 2D). The presence of nuclear FABP7 immunoreactivity in grades II, III, and IV astrocytoma specimens was statistically significant when compared to grade I pilocytic astrocytoma (Table 1).

**Figure 3:**
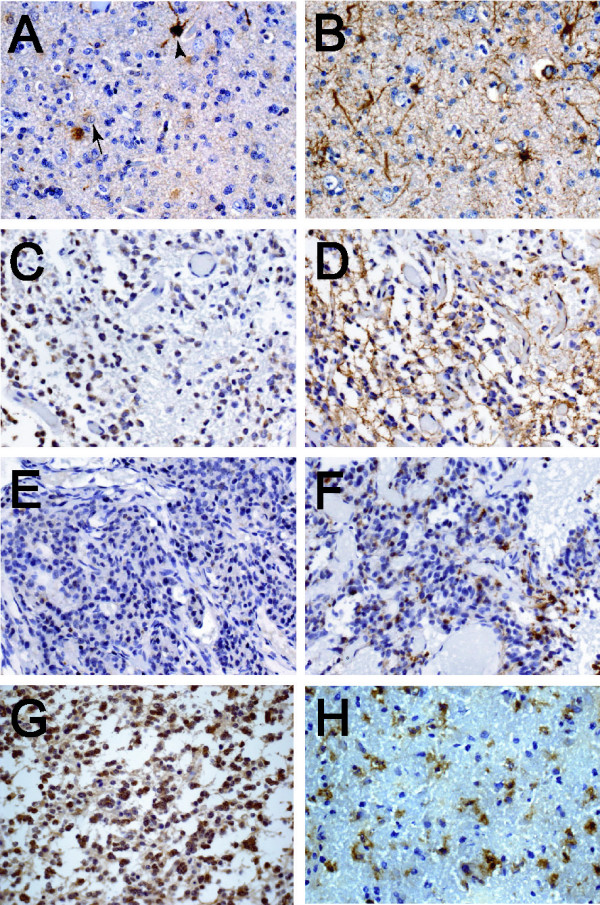
FABP7 is expressed in GFAP-positive cells in ODG and OAC. In grade III ODG, FABP7 (*A*) was expressed only in a subset of reactive astrocytes resembling Types 3 (arrow) and 4 (arrowhead) cells when compared to numerous GFAP-positive cells in an adjacent section (*B*). FABP7 was expressed in both nuclei and cytoplasm of the microgemistocytes (*C*) that were GFAP-immunoreactive (*D*). However, some tumors with microgemistocytes clearly did not express detectable amount of FABP7 (*E*) compared to GFAP (*F*). FABP7 was also seen in both nuclei and cytoplasm of tumor cells in some grades II (*G*) and III (*H*) OAC, and adjacent sections showed that the same groups of cells were GFAP positive (data not shown). The scale of the photomicrographs is the same as in Figure 1 *A*.

Among 19 grade III ODG specimens examined, FABP7 immunoreactivity was seen mostly in cells that were morphologically similar to Types 3 and 4 cells (Fig. 3A and 3B) and appeared to be reactive astrocytes. Neoplastic oligodendrocytes did not express FABP7. Similar to gliotic brain tissue, only a fraction of reactive astrocytes marked by GFAP staining expressed FABP7. FABP7 could also be detected in the nuclei and cytoplasm of microgemistocytes in some ODG specimens (Fig. 3C and 3D). Microgemistocytes are a group of neoplastic cells with eosinophilic cytoplasm and GFAP immunoreactivity that are sometimes present in ODG; however, half of our specimens (3 of 6) that contained GFAP-positive microgemistocytes did not express appreciable amounts of FABP7 (Fig. 3E and 3F). We found both cytoplasmic and nuclear FABP7 immunoreactivity present in 4 of 7 grade II (Fig. 3G) and 8 of 10 grade III OAC specimens (Fig. 3H), and those FABP7-immunoreactive specimens were also positive for GFAP (data not shown).

**Table 1:** Summary of subcellular localization of fatty acid binding protein 7 in gliomas

Diagnosis	Total Number examined	nuclear only or nuclear/cytoplasmic positive	Cytoplasmic positive only	negative	p value^1^
grade I pilocytic astrocytoma	10	0	9	1	NA^2^
grade II astrocytoma	5	1	0	4	0.003
grade III astrocytoma	5	3	0	2	0.001
grade IV GBM	9	3	3	3	0.022
grade II oligoastrocytoma	7	2	2	3	0.027
grade III oligoastrocytoma	10	5	3	2	0.006
grade III oligodendroglioma	19	3	0	16	NA^3^

^1 ^The significance of nuclear FABP7 between 9 FABP7-positive grade I pilocytic astrocytoma specimens and the other type of FABP7-positive tumors was evaluated using Mann-Whitney test.

^2 ^Minimal number of FABP7-positive nuclei were occasionally seen in all grade I tumors and we did not consider them for statistics.

^3 ^FABP7 was not expressed in neoplastic oligodendrocytes.

**Table 2:** A set of 61 GBM specimens for analyses of patient survival and the correlation between FABP7 and EGFR expression

Case #	Diagnosis	Age	Survival (wk)	Censor	FABP7 Nu	FABP7 Cyto	New Category*	EGFR IHC	P53 IHC	MIB-1
160	GBM	29	253	1	0	0	0	0	0	16.3
381	GBM	32	220	1	0	0	0	0	3	32.6
259	GBM	35	412	1	0	2	0	0	3	5.9
495	GBM	38	125	1	0	2	0	0	3	2.2
262	GBM	39	75.571	1	0	1	0	0	3	12.7
322	GBM	40	33.571	1	0	0	0	0	3	14.7
483	GBM	41	18.4286	1	0	1	0	0	1	6.2
499	GBM	43	26.143	1	0	0	1	2	1	10.8
198	GBM	43	83	1	0	1	0	0	3	32.7
310	GBM	43	66.143	1	0	2	0	0	0	33.6
307	GBM	44	65.4286	1	0	1	0	0	3	29.1
497	GBM	44	138.429	1	0	1	0	0	0	62.1
436	GBM	45	151.286	1	0	0	0	0	1	6.6
212	GBM	46	106.571	1	0	0	0	0	2	39.6
208	GBM	49	68.429	1	0	1	0	0	1	23.1
8	GBM	52	45.429	1	0	2	1	1	1	5.9
501	GBM	53	115.571	1	0	0	0	0	3	47.5
330	GBM	53	20.7143	1	0	1	0	0	2	36.5
446	GBM	56	41.571	1	0	0	0	0	3	39.2
404	GBM	56	68.857	1	0	2	0	0	3	36.1
288	GBM	57	38.2	1	0	0	1	1	2	21.8
19	GBM	58	80.429	1	0	2	0	0	3	6.1
292	GBM	58	38.2	1	0	2	NA	NA	0	NA
472	GBM	60	36.714	1	0	0	0	0	0	52.6
286	GBM	61	51.286	1	0	2	0	0	1	51.3
341	GBM	61	39.286	1	0	2	0	0	3	75.2
135	GBM	63	21.143	1	0	2	0	0	1	44.6
494	GBM	63	47.2	1	0	2	1	1	NA	NA
426	GBM	64	83.143	1	0	2	0	0	3	34.6
295	GBM	65	58.429	1	0	2	0	0	3	22.1
260	GBM	67	22.5	1	0	0	NA	NA	0	11.7
329	GBM	67	58.5	1	0	2	NA	NA	NA	NA
321	GBM	69	24.571	1	0	0	0	0	1	46.9
298	GBM	72	22.429	1	0	0	0	0	1	27.5
309	GBM	76	29.429	1	0	0	0	0	1	91.9
409	GBM	15	149.714	1	1	2	2	0	3	25.4
254	GBM	25	101.571	1	1	2	2	0	3	34.8
403	GBM	35	58	1	1	2	2	0	1	37.9
192	GBM	51	36	1	1	1	2	0	1	31.7
240	GBM	53	8.143	1	1	2	2	0	3	49.2
124	GBM	54	46.143	1	1	2	2	0	1	41.7
470	GBM	56	81	1	1	2	NA	NA	0	35.5
408	GBM	57	44	1	1	1	2	0	1	32.8
425	GBM	57	15.143	1	1	1	3	1	1	31.3
279	GBM	59	47.143	1	1	2	3	1	2	23.4
493	GBM	68	49.5	1	1	2	NA	NA	NA	NA
471	GBM	70	42.286	1	1	1	3	2	0	25.5
496	GBM	70	55.857	1	1	1	3	2	0	21.8
245	GBM	70	22.5	1	1	2	NA	NA	2	22
241	GBM	32	33.143	1	2	0	2	0	3	25.6
261	GBM	33	21.571	1	2	2	2	0	2	50.1
297	GBM	43	107.571	1	2	2	3	1	2	32.9
323	GBM	46	81	1	2	2	2	0	1	16.9
263	GBM	47	33.286	1	2	2	3	2	3	17.8
90	GBM	48	66.857	1	2	1	3	2	1	15.4
230	GBM	50	17.714	1	2	2	2	0	1	35.8
227	GBM	52	53.1429	1	2	2	3	1	1	43.1
325	GBM	52	32.143	1	2	2	3	2	0	20.3
443	GBM	55	59.286	1	2	1	3	2	1	39.7
296	GBM	59	39	1	2	0	2	0	1	44.9
335	GBM	75	18.143	1	2	2	2	0	1	27.5

*Based on nuclear FABP7 and EGFR immunoreactivity: dual negative as "0", nuclear FABP7-/EGFR+ as "1", nuclear FABP7+/EGFR- as "2", and dual positive as "3"

NA, no IHC data available.

### Nuclear fatty acid-binding protein 7 in glioblastoma is associated with epidermal growth factor receptor overexpression

Increased immunoreactivity of nuclear FABP7 correlated with poor prognosis of GBM, particularly in younger patients [9], and we sought to identify the molecular mechanism underlying this association by investigating EGFR expression in this tumor type.

**Figure 4:**
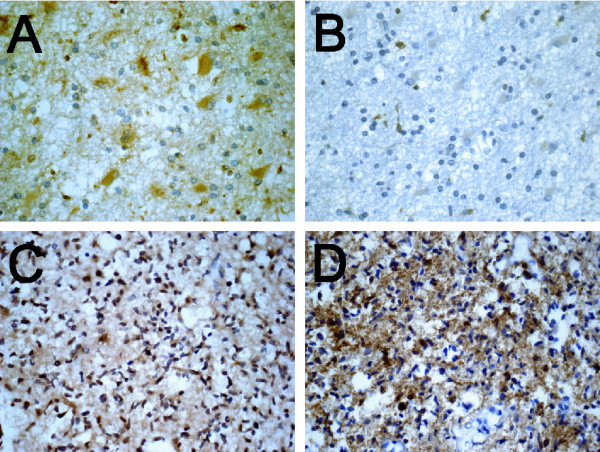
EGFR expression is associated with nuclear but not cytoplasmic FABP7 immunoreactivity. In one region of a representative GBM specimen, the cytoplasm of neoplastic gemistocytes had pronounced FABP7 immunoreactivity but the nuclei of the same cells were negative (*A*). In an adjacent section of the same specimen only minimal EGFR staining was seen in the same population of tumor cells (*B*). In another region of the same specimen, all nuclei of neoplastic astrocytes were marked by FABP7 staining (*C*), and EGFR expression was also prominent in these cells (*D*). The scale of photomicrographs is the same as in Figure 1 *A*.

EGFR expression evaluated by immunohistochemistry using a group of primary GBM specimens from 61 patients (Table 2) showed a trend toward a negative association with survival (p = 0.074), which was marginally correlated only in younger patients (less than the median age of 53 years; p = 0.037, hazard ratio = 1.819, 95% CI 1.036–3.195) but not in the old ones (p = 0.875), consistent with the previous finding [7]. Since we noted a similar trend for nuclear FABP7 staining in this same set of specimens [9], it raised the possibility of a correlation between EGFR expression and nuclear FABP7. Indeed, EGFR expression correlated with nuclear FABP7 (p = 0.008) but not with cytoplasmic FABP7 (p = 0.547) in this set of specimens. This finding is consistent with a detailed examination of FABP7/EGFR dual-positive GBM specimens with separate regions of nuclear and cytoplasmic FABP7 immunoreactivity. We found that if a cluster of tumor cells had only cytoplasmic FABP7 immunoreactivity, the EGFR staining was minimal (Fig. 4A and 4B). In contrast, when neoplastic cells in another region of the same tumor had nuclear FABP7 immunoreactivity, the EGFR immunoreactivity was usually prominent (Fig. 4C and 4D). The significant association between nuclear FABP7 and EGFR immunoreactivity seemed to be present only when the entire population, not specific age group, was analyzed, since the correlation was greatly reduced in both younger (p = 0.048, N = 31) and older groups (p = 0.055, N = 27) divided by the median age (53 years) of the cases.

The link between EGFR and nuclear FABP7 appeared to be specific for the neoplastic cells in GBM since nuclear FABP7 immunoreactivity did not co-exist with EGFR expression in several other types of specimens (data not shown). These were: (1) FABP7-positive microgemistocytes in ODG, (2) FABP7-positive reactive astrocytes in morphologically normal brain regions surrounding the EGFR-positive GBM, (3) FABP7-positive reactive astrocytes in gliotic tissues, and (4) Type 1 cells in normal cerebrum. Likewise, EGFR-positive cells surrounding the EGFR-overexpressing GBM that morphologically resembled reactive astrocytes did not show FABP7 immunoreactivity (data not shown).

**Figure 5:**
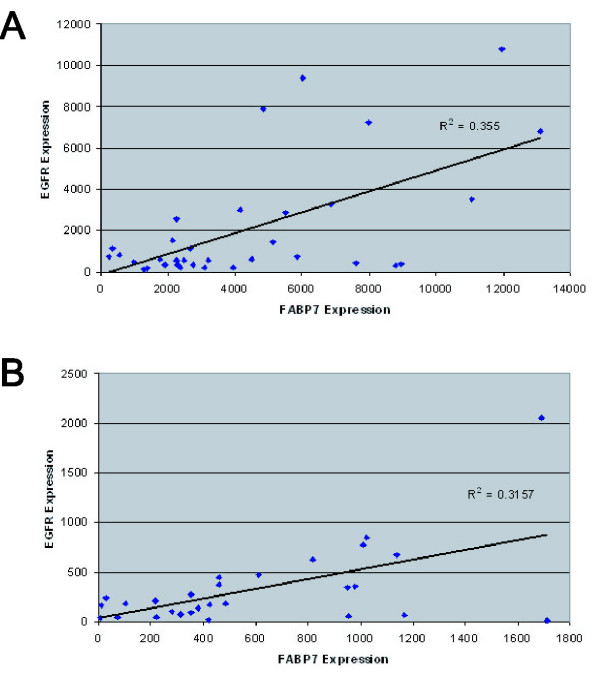
Scattered plot analyses of the expression levels of *FABP7 *and *EGFR *genes. The abundance of mRNA for both genes obtained by microarrays was presented on a log2 base. A strong positive correlation of *FABP7 *and *EGFR *expression was seen in a set of 34 GBM specimens (*A*, p = 0.016), which was validated by a public microarray dataset using 28 GBM specimens (*B*, p = 0.03).

We further examined whether the association between nuclear FABP7 and EGFR expression is reflected on the transcription level of both genes in two independent published DNA microarray datasets (Table 3). Among the 34 GBM specimens analyzed by oligonucleotide microarrays [29], mRNA expression of both *FABP7 *and *EGFR *genes moderately correlated with each other (Fig. 5A, p = 0.016). In another report using 28 GBM specimens with the same microarray system [30], expression of both genes once again correlated with each other (Fig. 5B, p = 0.03). This observation is in agreement with the marginal correlation between EGFR and the combination of both nuclear and cytoplasmic FABP7 immunoreactivity in our 61 specimens (p = 0.033).

**Table 3:** Expression of FABP7 and EGFR mRNA in GBM from two independent published DNA microarray datasets

**Dataset 1 (ref. 29)**			**Dataset 2(ref. 30)**		
	
Case #	FABP7	EGFR	Case #	FABP7	EGFR
	
47.0	227.5	763.5	1375	28.8	233.8
210.4	337.0	1113.8	276	1010.0	775.2
193.0	555.6	831.6	519	72.1	46.7
21.7	974.6	471.4	368	418.2	14.7
97.3	1277.9	146.0	157	6.3	35.2
147.9	1384.4	188.3	1162	378.0	139.9
365.0	1758.1	625.4	1644	9.8	165.8
131.7	1908.4	367.4	406	950.8	341.8
459.4	2126.0	1522.5	308	611.3	473.7
33.9	2246.2	2588.0	177	817.8	630.8
58.7	2250.7	558.9	103	313.2	74.4
158.0	2289.3	327.5	992	1024.3	842.2
22.3	2351.9	224.2	41	952.3	56.6
97.9	2480.0	578.6	1354	101.3	182.9
14.0	2662.4	1134.1	308	424.0	169.2
205.9	2746.3	357.6	408	279.7	97.3
232.0	3071.7	220.9	242	1137.2	673.9
226.6	3190.2	547.5	323	218.8	208.4
29.4	3944.2	196.8	213	219.6	49.0
68.0	4158.9	3010.9	97	352.8	87.4
5.3	4496.3	625.6	281	978.6	357.0
31.1	4831.9	7883.7	501	1166.7	64.5
83.0	5120.5	1440.0	670	461.1	444.7
80.0	5508.2	2871.1	729	1711.5	8.9
82.0	5854.7	741.7	21	1690.3	2057.1
103.6	6008.9	9373.4	630	353.7	274.0
2.6	6850.0	3259.5	263	460.3	375.6
41.3	7593.1	441.2	219	486.4	179.7
60.9	7966.5	7258.6			
46.1	8795.5	284.8			
40.3	8946.3	410.0			
58.3	11070.1	3518.5			
50.4	11956.1	10788.3			
16.3	13108.3	6798.5			

**Table 4:** An independent set of 44 GBM specimens to examine the effects of nuclear FABP7 and EGFR expression on patient survival

Case #	Diagnosis	Age	Survival (wk)	Censor	FABP7 Nu	EGFR IHC	New Category*
17	GBM	54.2	73.2	1	0	0	0
30	GBM	24.3	203.9	1	0	0	0
45	GBM	45.2	67.9	1	0	0	0
46	GBM	26.9	146.7	0	0	0	0
66	GBM	17.5	133.5	0	0	0	0
68	GBM	31.6	146.9	1	0	0	0
81	GBM	51.1	60.6	1	0	0	0
84	GBM	43.6	66.8	0	0	0	0
87	GBM	59.9	43.4	1	0	0	0
91	GBM	46.4	383.0	0	0	0	0
94	GBM	54.0	33.7	1	0	0	0
98	GBM	39.1	48.6	1	0	1	1
99	GBM	56.9	128.1	1	0	1	1
107	GBM	59.1	79.8	1	0	2	1
110	GBM	45.2	35.1	1	0	2	1
148	GBM	59.8	35.1	1	0	2	1
194	GBM	54.8	32.7	1	0	2	1
225	GBM	54.1	94.3	1	0	2	1
243	GBM	50.6	76.8	1	0	2	1
263	GBM	54.7	123.1	1	0	2	1
277	GBM	42.5	25.1	0	0	2	1
284	GBM	47.7	92.3	1	0	2	1
29	GBM	35.3	97.0	1	0	2	1
89	GBM	55.2	45.1	1	1	0	2
273	GBM	42.5	39.0	0	1	0	2
4	GBM	51.0	29.5	1	1	0	2
21	GBM	59.3	32.7	1	1	0	2
32	GBM	58.2	22.1	1	1	0	2
53	GBM	51.2	23.8	1	2	0	2
54	GBM	33.1	32.7	0	2	0	2
58	GBM	37.2	31.1	1	2	0	2
61	GBM	12.8	12.3	0	2	0	2
88	GBM	52.2	93.9	1	2	0	2
100	GBM	45.8	11.3	1	2	0	2
103	GBM	55.1	13.9	1	2	2	3
105	GBM	41.7	46.1	1	2	2	3
106	GBM	43.8	10.5	0	2	2	3
207	GBM	51.3	27.1	1	2	2	3
240	GBM	45.2	13.3	1	2	2	3
247	GBM	58.7	43.3	1	2	2	3
265	GBM	58.8	80.1	1	2	2	3
34	GBM	54.4	36.0	1	1	1	3
38	GBM	23.0	24.1	1	1	2	3
50	GBM	25.0	41.0	1	1	2	3

*Based on nuclear FABP7 and EGFR immunoreactivity: dual negative as "0", nuclear FABP7-/EGFR+ as "1", nuclear FABP7+/EGFR- as "2", and dual positive as "3"

### The presence of nuclear FABP7 immunoreactivity in EGFR-expressing GBM specimens is associated with shorter survival compared to those negative for nuclear FABP7

In our 61 GBM specimens poor survival was associated with increased nuclear FABP7 immunoreactivity as well as EGFR expression. Our data also showed that expression of *FABP7 *gene and nuclear FABP7 immunoreactivity correlated with mRNA and protein expressed by *EGFR*, respectively. Therefore, we hypothesized that nuclear FABP7 and EGFR expression might have additive association with poor prognosis.

**Figure 6:**
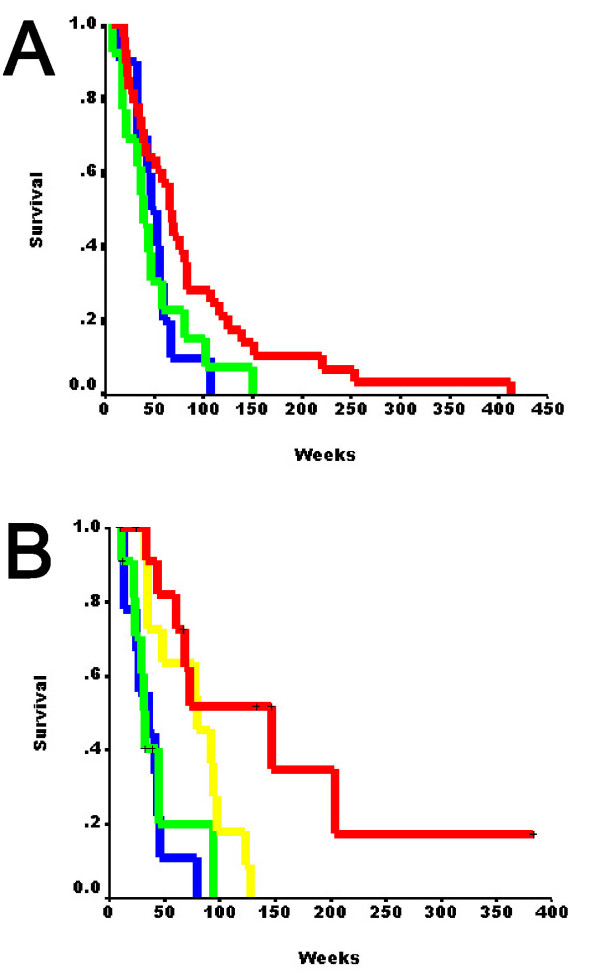
Kaplan-Meier analysis of the first set of 55 GBM patients based on the new   categories (0, N = 28; 1, N = 4; 2, N = 13; 3, N=10) of nuclear FABP7 and   EGFR expression (see Methods) showed that dual-negative specimens had   significant longer survival time (*A*). Kaplan-Meier analysis of an   independent set of 44 younger GBM patients (0, N = 11; 1, N = 12; 2, N = 11;   3, N=10) showed gradual decrease of survival time based on increased   expression of nuclear FABP7 and EGFR (*B*). Red, category 0; yellow, category   1; green, category 2; blue, category 3.

We examined the association of nuclear FABP7 with patient survival in relation to the EGFR expression in the 55 cases (Table 2) that had all parameters available using newly designated variables as defined in Methods: dual negative as "0", nuclear FABP7-negative/EGFR-positive as "1", nuclear FABP7-positive/EGFR-negative as "2", and dual positive as "3". Cox regression analysis of these cases (category 1 had only 4 cases and thereby was excluded) showed that, although nuclear FABP7-positive tumors (combining categories 2 and 3) demonstrated negative association with survival (p = 0.022, hazard ratio = 1.408, 95% CI 1.05–1.888), the survival curves between specimens of the categories 2 and 3 were indistinguishable (Figure 6A, p = 0.509). However, we could not determine the interaction between EGFR expression and nuclear FABP7 immunoreactivity in the survival of GBM patients since there were no enough cases from category 1.

We analyzed another group of 44 GBM specimens used in our earlier study [9] that had more category 1 specimens (N = 12, Table 4). Consistent with what was observed in the previous set, cases from categories 2 and 3 together had significantly shorter survival compared to those of category 0 (p = 0.001, hazard ratio = 5.604, 95% CI 1.985–15.821), but showed no difference from each other (Figure 6B, p = 0.692). Cox regression analysis showed that the category 1 cases had shorter survival than immuno-negative tumors but better prognosis compared to the category 3 specimens (p = 0.009, hazard ratio = 4.167, 95% CI 1.421–12.222), suggesting that detection of nuclear FABP7 immunoreactivity might predict poor prognosis for EGFR-positive GBM.

### Epidermal growth factor treatment increases immunoreactivity of fatty acid-binding protein 7 in nucleus

To investigate whether EGFR activation-induced FABP7 expression observed in Schwann cells [13] is present in GBM, we examined FABP7 expression and its subcellular localization upon EGFR activation. Four commonly used EGFR-expressing glioma cell lines, U87, U251, SF763, and SF767, were examined for FABP7 expression using immunohistochemistry, and all these lines demonstrated both cytoplasmic and nuclear FABP7 immunoreactivity (data not shown). The SF763 glioma cell line was chosen for this study because it had the lowest ratio of nuclear to cytoplasmic FABP7 as compared to other cell lines, and induction of FABP7 nuclear translocation by EGFR activation could be easily detected. 

**Figure 7:**
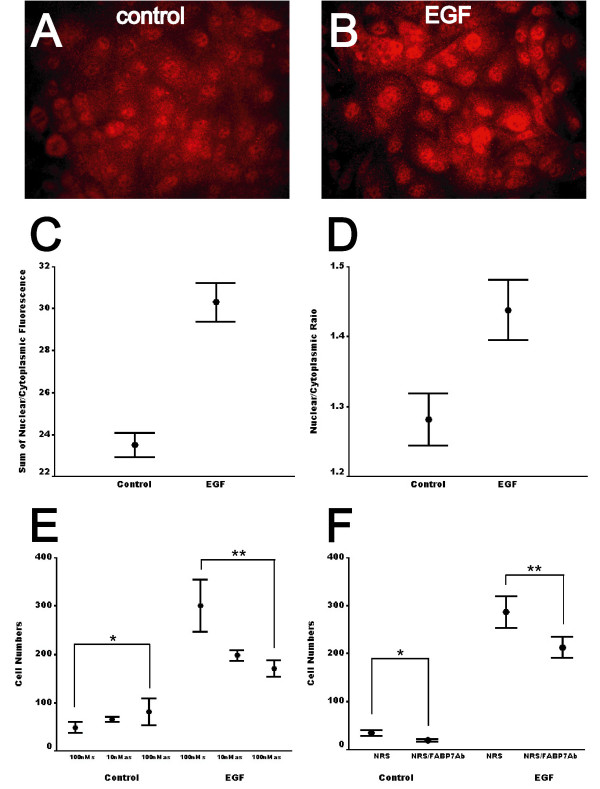
EGF activation induces nuclear translocation of FABP7 in glioma cells. Immunofluorescent staining of FABP7 in SF763 glioma cells displayed both cytoplasmic and nuclear immunoreactivity (*A*), whereas FABP7 staining in the nuclei was increased after EGF treatment for 2 days (*B*). The scale of photomicrographs is the same as in Figure 1A. *C*, the sum of nuclear and cytoplasmic FABP7   fluorescence in SF763 cells in each of the 10 randomly chosen fields was   calculated, and EGF treatment significantly increased total FABP7   fluorescence. *D*, the ratios of nuclear/cytoplasmic FABP7 in untreated SF763   glioma cells and cells treated with 50 ng/ml of EGF for 1 day were   semi-quantitatively measured. For each group, ratios of 20 cells were   calculated from each of the 10 randomly chosen fields (p < 0.001). *E*,   control SF763 cells demonstrated no difference in migration in the presence   of either FABP7-specific sense or antisense ODNs (*, p = 0.136), whereas   migration of EGF-treated SF763 cells was reduced by more than one-third in   the presence of antisense ODNs (**, p = 0.004). *F*, FABP7 polyclonal   antibodies also inhibited migration of both control and EGF-treated SF763   cells (*, p=0.003; **, p=0.01). Error bars represented the standard errors   of the mean.

In SF763 cells, FABP7 expression increased after EGF treatment (Fig. 7A and 7B). To avoid artifacts caused by the heterogeneity of immunostaining or the increased expression of FABP7 after EGFR activation, the ratio of nuclear to cytoplasmic FABP7 immunoreactivity of each cell was calculated. The nuclear to cytoplasmic ratio of FABP7 staining in EGF-treated SF763 cells was statistically higher than in the group without EGF treatment (p < 0.001, Fig. 7C), suggesting that nuclear translocation of FABP7 in GBM could be induced by EGFR activation.

### Epidermal growth factor-induced glioma cell migration is suppressed by inhibiting fatty acid binding protein 7 expression

Since both EGFR activation [31] and forced expression of FABP7 [9] promote glioma cell migration, we examined whether inhibiting the expression of FABP7 can suppress glioma cell migration induced by EGF.

Antisense oligodeoxynucleotides (ODNs) have been successfully used to reduce expression of other members of the FABP family [32], so we examined whether inhibiting FABP7 expression with ODNs affects glioma cell migration. We found that FABP7-specific antisense ODNs suppressed more than one-third of EGF-induced SF763 cell migration, but had no effect on migration of the cells without EGF treatment (Fig. 7D). This result strongly suggests that at least a portion of EGF-induced glioma cell migration is mediated through FABP7.

## Discussion

In previous work, we identified nuclear FABP7 immunoreactivity as a prognostic marker for patients with GBM [9]. In a separate report, increased expression of FABP7 was also found in GBM patients surviving less than 2 years compared to those who survived longer [29]. Two cellular functions for FABP7 have been identified: cell migration and differentiation in the developing central nervous system [11], and Schwann cell-axonal interactions in the peripheral nervous system [13]. Although suggestive, neither offers a clear explanation of the action of FABP7 in gliomas. The goals of this study were to determine a plausible biological role for FABP7 in glioma pathogenesis. We characterize the expression of FABP7 in normal brain, gliotic tissues, and glial tumors. We show that increased FABP7 expression occurs in a subset of reactive astrocytes, that FABP7 expression is restricted to cells of astrocytic lineage in glioma, and that there is almost no nuclear FABP7 immunoreactivity in well-circumscribed pilocytic astrocytoma. In addition, we establish an association between nuclear FABP7 and EGFR expression both in human GBM tumors and in a glioma cell line. Our data suggest that FABP7 might play a role in GBM pathogenesis through its participation in the EGFR signaling pathways. Most importantly, we identify nuclear FABP7 immunoreactivity as a marker to predict the outcome of patients with EGFR-positive GBM.

Transcription of both *FABP7 *and *EGFR *genes showed marginal association based upon the microarray datasets, but only nuclear FABP7 immunoreactivity correlated with EGFR expression. Age is one of the most important prognostic factors for GBM patients, and it has been shown in separate studies that the status of EGFR expression is a better prognostic factor in younger patients [7,33]. Our data indicate that the prognostic value of nuclear FABP7 is also influenced by patient age [9]. However, the correlation between nuclear FABP7 and EGFR expression did not have preference to patient age in our study. Collectively, it appears that other factors (age is probably one of them, see below) yet to be identified participate in regulating the transcriptional and translational mechanisms shared by both *FABP7 *and *EGFR *genes, as well as in regulating the subcellular localizations of FABP7. This is compatible with the fact that EGFR expression and nuclear FABP7 immunoreactivity are mutually exclusive in a significant portion our clinical specimens. It will be pressing to define in the future why nuclear FABP7 is not present in some EGFR-positive GBM and whether other EGFR-downstream pathways are active in EGFR-negative/nuclear FABP7-positive tumors.

It has been previously reported that EGFR signaling induces FABP7 expression in a Ras-independent pathway in normal and tumor Schwann cells [13], providing evidence of possible interaction between these two proteins. Our demonstration in GBM specimens that expression of both FABP7 and EGFR correlated with each other at both protein and mRNA levels, and in SF763 glioma cells that EGFR activation induced nuclear translocation of FABP7, warrants further investigation of whether FABP7 is a direct downstream target of EGFR activation in GBM and which EGFR pathway FABP7 is associated with. It has been shown that liver-type FABP carries fatty acids to interact with nuclear receptors that in turn regulate gene expression [22-24]. If FABP7 utilizes a similar mechanism to regulate gene expression in response to EGFR activation, this would expand the scope of EGFR signaling pathways in GBM tumors. Future studies will examine the specific molecular pathways linking EGFR and FABP7, and nuclear functions of FABP7.

In normal cerebral cortex, we identify a unique population of glia positive for FABP7 but negative for GFAP that we designated as Type 1 cells. The origin and exact role of these cells remains unclear. NG2, a chondroitin sulfate proteoglycan, is expressed by oligodendrocyte progenitors, and identification of one type of GFAP-negative/NG2-positive astrocytes in adult normal brain and a subset of gliomas led to a hypothesis that certain gliomas arise from the NG2-positive progenitor cells [34]. According to the morphology and frequency of appearance [35], Type 1 cells identified in this study clearly do not belong to this category. However, based upon the expression patterns of FABP7 during the development of central nervous system, in adult brain, and in gliomas, transformation of FABP7-positive cells may contribute to the histogenesis of a subgroup of gliomas.

In gliotic brain tissue, FABP7 expression is increased in a subset of reactive astrocytes and demonstrates variable subcellular localization in the cytoplasm and nucleus. Such differential patterns in both expression and subcellular localization of FABP7 are also seen in cells with astrocytic features in various types of glioma. As noted above, grade I pilocytic astrocytoma is the only type of glioma examined that does not show nuclear FABP7 staining in our studies. Although our size of sampling was limited, the statistical analyses clearly demonstrate that the chance of detecting nuclear FABP7 in pilocytic astrocytoma is small. Because pilocytic astrocytomas are well-demarcated lesions whose pattern of growth is clearly distinctive from diffusely infiltrative higher-grade (grades II to IV) astrocytoma and oligodendroglial tumors (both ODG and OAC), considering the association of nuclear FABP7 with poor prognosis of GBM patients, cytoplasmic localization of FABP7 may be associated with less infiltrative phenotype of neoplastic astrocytes. Interestingly, we did find positive correlation between cytoplasmic FABP7 immunoreactivity and patient survival in cases older than the medium age among the first set of 61 GBM specimens (data not shown), although this observation has not been validated in an independent set of samples. It would be important in the future to investigate the downstream signaling pathways for FABP7 in the nucleus and cytoplasm and their association with cell motility, and how the translocation of FABP7 is regulated, especially under the context of patient age.

Although FABP7 expression does not predict the outcome of patients with ODG and OAC [36], FABP7 may have prognostic value for grade II and grade III astrocytomas due to its heterogeneous patterns of expression and subcellular localization in these two tumor types. In other experiments, we noted that immortalized non-tumorigenic astrocytes express similar amounts of FABP7 compared to glioma cell lines (data not shown). FABP7 overexpression in glioma cells does not affect cell cycle progression and activation of apoptosis [9]. In gliotic brain tissue increased FABP7 expression coincides with GFAP expression in a subset of reactive astrocytes. Increased expression of FABP7 was seen in brain tissues after systematic administration of a neurotoxin, kainic acid [14]. FABP7 was released into patients' serum after acute ischemic stroke [37]. For these reasons, FABP7 expression or the presence of nuclear FABP7 alone is unlikely a factor unique to glioma oncogenesis and progression. On a practical level, however, anecdotal experience suggests that occasional cases exist where the main differential diagnosis is pilocytic astrocytoma versus GBM (both of these astrocytic tumors exhibit microvascular proliferation), and the presence of nuclear FABP7 would support the diagnosis of GBM.

In addition to this report, elevated levels of FABP7 mRNA and protein in GBM specimens compared to those in normal adult brain have been previously demonstrated [9,12]. Opposite results were reported in breast and prostate cancer, where decreased expression of FABP7 was found in tumor specimens when compared to normal tissues [19,32]. Greater FABP7 expression was seen in rarely metastasized melanoma cell lines compared to their frequently metastasizing counterparts [38]. Poorly-differentiated prostate tumors lose FABP7 expression, but more FABP7 is expressed in well-differentiated prostate cancer specimens than in primary normal prostate cells [32]. Ectopic expression of FABP7 induces cell differentiation and suppresses tumor growth [19,20] and was shown to mediate the cytotoxicity of DHA to breast cancer cells [20]. One explanation for these divergent results in patterns of expression and effects of forced expression is that functions of FABP7 in neoplastic and normal cells are tissue-specific. A strong candidate factor involved in such tissue-specific functions is EGFR, since we observed in clinical specimens the correlation of gene expression between *FABP7 *and *EGFR*, and the correlation of nuclear FABP7 immunoreactivity with EGFR expression; however, this association appears to be only in GBM, but not in normal brain, gliotic tissues, or other types of glioma examined.

Our immunohistochemical results show the change of subcellular localization of FABP7 in normal, gliotic, and neoplastic brain tissues and suggest several interesting avenues for future studies. Based on its amino acid sequence and protein structure, the primary activity of FABP7 appears to be binding of fatty acids. Although the binding affinity of FABP7 for various types of long-chain fatty acids has been studied *in vitro*, and a likely *in vivo *ligand for FABP7 has been proposed [15], the actual fatty acids bound to FABP7 are yet to be determined. Because FABP7 does not possess an obvious nuclear localization signal, nuclear translocation of FABP7 might require other carrier proteins. Defining these interacting proteins and ligands should clarify the biological roles of FABP7.

## Conclusion

FABP7 is preferentially expressed in cells of astrocytic features, and demonstrates variable expression levels and subcellular localization in gliotic tissues and all grades of astrocytoma, indicating that FABP7 expression alone does not contribute to malignant progression of astrocytomas. However, nuclear localization of FABP7 may be associated with infiltrative phenotype of glioma cells and EGFR pathways based on several findings: correlation of nuclear FABP7 immunoreactivity with poor prognosis of younger patients with GBM, association with EGFR expression in GBM, lack of nuclear FABP7 immunoreactivity in grade I astrocytomas, and induction of translocation of FABP7 into nucleus in glioma cells after EGFR activation.

## Abbreviations

FABP7, brain-type fatty acid-binding protein; GBM, glioblastoma; EGFR, epidermal growth factor receptor; DHA, docosahexaenoic acid; GFAP, glial fibrillary acidic protein; ODN, oligodeoxynucleotide; CI, confidence interval.

## Competing interests

The author(s) declare that they have no competing interests.

## Authors' contributions

YL designed the study, performed the experiments, analyzed the data, and wrote the manuscript. AWB and KDA participated in evaluating the histopathology and immunohistochemistry of the specimens. NG participated in analyzing the data and critically editing the manuscript. All authors read and approved the final version of the manuscript.

## Pre-publication history

The pre-publication history for this paper can be accessed here:


